# Design and analysis of individually randomized multiple baseline factorial trials

**DOI:** 10.3758/s13428-025-02874-1

**Published:** 2026-01-05

**Authors:** Yongdong Ouyang, Maria Laura Avila, Anna Heath

**Affiliations:** 1 Department of Biostatistics and Bioinformatics, Roswell Park Comprehensive Cancer Center, Elm and Clarton St, Buffalo, NY 14263 USA; 2 Roswell Park Graduate Division, University at Buffalo, Buffalo, NY 14260 USA; 3 Child Health Evaluative Sciences, The Hospital for Sick Children, Toronto, ON M5G 1E8 Canada; 4 Dalla Lana School of Public Health, University of Toronto, Toronto, ON M5S 1A1 Canada; 5 Department of Pediatrics, The Hospital for Sick Children, Toronto, ON M5G 1E8 Canada; 6 Department of Statistical Science, University College London, London, UK

**Keywords:** Multiple baseline, Randomized controlled trials, Rare diseases, Power, Small sample, Linear mixed effect model, Generalized estimating equations, Factorial design

## Abstract

Assessing the effectiveness of behavioral interventions in rare diseases is challenging due to extremely limited sample sizes and ethical challenges with withholding intervention when limited treatment options are available. The multiple baseline design (MBD) is commonly used in behavioral science to assess interventions, while allowing all individuals to receive the intervention. MBD is primarily used to evaluate a single intervention so an alternative strategy is needed when evaluating more than one intervention. In this case, a factorial design may be recommended, but a standard factorial design may not be feasible in rare diseases due to extremely limited sample sizes. To address this challenge, we propose the individually randomized multiple baseline factorial design (MBFD), which requires fewer participants but can attain sufficient statistical power for evaluating at least two interventions and their combinations. Furthermore, by incorporating randomization, we enhance the internal validity of the design. This study describes the design characteristics of a standard MBFD, clarifies estimands, and introduces three statistical models under different assumptions. Through simulations, we analyze data from MBFD using linear mixed effect models (LMM) and generalized estimating equations (GEE) to compare biases, sizes, and power of detecting the main effects from the models. We recommend using GEE to mitigate potential random effect misspecifications and suggest small sample corrections, such as Mancl and DeRouen variance estimator, for sample sizes below 120.

## Introduction

Conducting clinical trials for rare diseases presents unique challenges that complicate the traditional trial design and statistical analysis. One major difficulty is the small sample size, as rare diseases inherently affect a limited population, making it difficult to recruit enough participants to achieve sufficient statistical power (Partington et al., 2022). Additionally, in some cases where low-risk interventions are being tested, researchers may prefer a trial design in which all participants receive every intervention, rather than being assigned to separate treatment arms, to maximize the available data, account for individual variability, and allow access to potentially effective interventions in areas of high therapeutic need. These constraints necessitate innovative trial designs to ensure robust and reliable conclusions.

To address these challenges, researchers can turn to methodologies developed for rigorous inference in small populations, such as single-case experimental designs (SCEDs). SCEDs represent a family of designs that use repeated measurements to establish a stable baseline and demonstrate experimental control after an intervention is introduced, allowing for valid causal inferences even with a single participant (Epstein & Dallery, 2022; Krasny-Pacini & Evans, 2018; Smith, 2012). The multiple baseline design (MBD) is one of the most frequently used SCEDs in social and behavioral sciences (Baer et al., 1968; Coon & Rapp, 2018). In a standard MBD, all individuals begin in a control or SoC condition, and the intervention is then introduced at staggered times across different sequences (Watson & Workman, 1981). The timing of receiving intervention can either be randomized (experimental design) or non-randomized (quasi-experimental design) (Kratochwill & Levin, 2010; Levin & Ferron, 2021). However, when it is possible, randomization in MBD enhances the internal validity, reduces the potential confounding, and ensures that characteristics across the randomization units are more comparable across different baseline sequences.

Usually, individuals in an MBD are followed up over time, and multiple outcome measures are taken before and after the intervention to establish a stable estimate of the intervention effects. This approach is an extension of the simple before-and-after design and is a robust way of assessing the effectiveness of the intervention as it rules out the potential impact of concurrent events that may be confounded with the intervention (Epstein & Dallery, 2022; Hawkins et al., 2007). This design involves both within- and between-sequence comparisons (Kennedy, 2022). An MBD assumes that if similar effects are observed before and after treatment initiation in different sequences, then this effect of treatment is unlikely to be a coincidence (Slocum et al., 2022).

When randomization is used, MBD may have several advantages over the conventional parallel-arm trial design, which compares an intervention against the SoC. Firstly, individuals receive both the SoC and the intervention, critical to avoid ethical issues around withholding a low-risk, potentially beneficial intervention (Binik, 2019). An MBD design may also boost recruitment as individuals who are interested in the intervention may only participate if they are assured of receiving the intervention at some point. Critically for rare diseases, MBD may also be the only design that provides sufficient statistical power given the available sample size (Sundin & Crespi, 2022).

Sometimes, investigators are interested in testing multiple interventions simultaneously against a SoC (Dziak et al., 2012). In this case, a factorial design is a common choice (Cipriani & Barbui, 2013). However, a standard factorial design does not share the same advantages as an MBD and requires a strong assumption that the interventions are independent (i.e., no interaction effects) to achieve maximum efficiency. Therefore, a standard factorial design may not always be ideal or even feasible, especially when the underlying disease has low prevalence.

A design that combines features of both factorial and MBD is available for evaluating cluster-level interventions, known as a stepped wedge factorial design (Hemming et al., 2015; Hussey & Hughes, 2007; Lyons et al., 2017; Ouyang et al., 2022). However, a factorial MBD that includes individual-level randomization and interventions has not been presented. Therefore, we propose a novel individually randomized multiple baseline factorial design (MBFD) to extend these ideas for individual-level interventions.

As a motivating example, deep vein thrombosis (DVT) is rare but the most common type of venous thrombosis in children (O’Brien et al., 2022; Raffini et al., 2009). The most common chronic complication of DVT is post-thrombotic syndrome (PTS) (M. L. Avila et al., 2016), which is a form of chronic venous insufficiency that manifests with edema, pain, and poor endurance in the affected limb. Compression therapy, considered as SoC, is the only management strategy for pediatric PTS (Mutlak et al., 2019). While it is effective at reducing PTS severity (L. Avila et al., 2021), poor adherence has been a concern (Avila et al., 2021; Montoya et al., 2016). Therefore, a strategy to improve adherence is needed. Compression therapy may also be unsuitable for some children, with one-third of the children finding compression garments too uncomfortable or too difficult to put on, or that their symptoms worsened with compression (Avila et al., 2021).

Drug interventions are gaining popularity for adults but are currently not used in pediatrics (Avila et al., 2024). Thus, trials of these medical interventions are needed in children. Since both an adherence-enhanced compression garment wear program and the use of pharmacological management may benefit children, we want to design a randomized trial to investigate the efficacy of individual and combined programs to decrease PTS symptom severity, measured by the CAPTSure score^©^ (Avila et al., 2019), a validated outcome measure in pediatric patients with lower limb PTS.

Since pediatric DVT is rare, there are inevitable recruitment challenges with a maximum recruitment for the study of around 30 patients. Given this restriction, the standard factorial trial would not provide sufficient statistical power to detect a treatment effect. Participants may also be unwilling to participate unless they are assured of receiving both new interventions at some point. Thus, we developed an individually randomized MBFD to address these two challenges and provide reasonable statistical power.

We critically examine and extend the design and analysis framework of MBFDs, with particular attention to their application in limited sample size settings such as rare diseases or early-phase interventions. We begin by formally defining the MBFD structure, emphasizing its deviation from traditional factorial and multiple baseline designs. We then clarify the estimands that can be unbiasedly identified under its constraints. Given the sequential exposure to treatments and the potential confounding from treatment order and carryover, we carefully consider the causal assumptions required to interpret effects, particularly when estimating combined treatment effects. We explore several statistical models tailored to this design, outlining their assumptions, limitations, and alignment with the estimands. A simulation study evaluates model performance under various conditions, including realistic intraclass correlation coefficients and empirically motivated treatment effect sizes, addressing concerns about generalizability and robustness. Finally, we discuss its broader implications, limitations, and avenues for future methodological development, particularly in improving causal interpretability and estimation efficiency.

## Design characteristics

### Standard multiple baseline factorial design

**Fig. 1:**
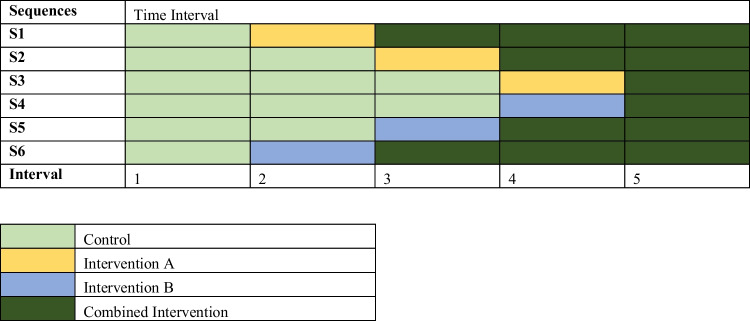
A standard multiple baseline factorial design in which individuals start in the control condition (*light green*) and then transition into either intervention A (*yellow*) or B (*blue*), depending on their randomization. Individuals spend one time interval receiving the single intervention, then transition into the combined intervention (*dark green*). Measurements are taken repeatedly at each time interval

Figure 1 introduces the standard MBFD to investigate the impact of two interventions (A and B) and their combination, which can be extended to more than two interventions. In this design, the outcome is repeatedly measured, with the number of outcome measurements and the time between outcome measurements selected to be sufficient to understand the variability in the outcome. Each column in Fig. 1 represents a time period. Each box in Fig. 1 represents a time interval in which the outcome is measured. The MBFD includes multiple treatment sequences that specify the number of intervals for which the patients will receive the control condition, the initial intervention, and the combination. We recommend a minimum of three sequences per intervention (Lanovaz & Turgeon, 2020), with everyone randomized to one of the sequences (rows in Fig. 1). After randomization, everyone begins the study receiving the control condition (light green cells). Following the initial treatment phase, individuals transition into either intervention A or B, and remain with the selected intervention for one interval before transitioning into the combined intervention. There are at least five intervals, and measurements are taken at the end of each interval for every individual. The standard MBFD design also ensures that everyone receives the combined intervention for one interval.

In our proposed MBFD, the combined intervention (AB) is always delivered sequentially following exposure to either A or B. As such, the AB effect should be interpreted as a conditional sequence effect. That is, the impact of receiving both interventions in sequence compared to SoC. This differs from the concurrent AB effect estimated in a traditional factorial trial. This sequential approach is particularly relevant in rare-disease settings where the sample size required for a pure factorial design is unattainable, and where maximizing exposure to potentially beneficial treatments is desirable. Our design differs from a conventional four-arm factorial design, in which four concurrent groups (e.g., control/SoC, A, B, and AB) are compared, and AB represents simultaneous delivery of both interventions. Such a structure allows direct estimation of main effects and the unconditional AB interaction. This choice to include a sequential combination was guided by the limited sample size available for rare disease trials and the desire to preserve the strengths of the MBD framework while exploring the effects of combined interventions.

Due to these limitations, the objective of our motivating trial is, therefore, to test the following three hypotheses:

1. A behavioral intervention to improve adherence (A) alone decreases PTS symptom severity compared to SoC.
2. Pharmacologic management of PTS (B) decreases symptom severity compared to SoC.
3. The sequential combination of A and B, following prior exposure to one of them, decreases PTS symptom severity compared to SoC.

### Extensions to standard multiple baseline factorial design

In Fig. 1, we demonstrate a standard design where all individuals receive the single intervention for a single time interval before transitioning to the combined intervention. This design can be modified for different applications. For example, if more than one outcome measurement is required to show intervention effects, then the number of intervals for the intervention can be extended. Secondly, the standard design assumes that all individuals exit the trial at the same time, which is convenient but individuals receive the combined intervention for a varied number of intervals. This can also be modified so that individuals leave the trial after receiving the combined intervention for a sufficiently long time. The number of sequences chosen is a crucial aspect of MBDs. The inclusion of more sequences can enhance the reliability of the conclusions (Lanovaz & Turgeon, 2020) and studies have also suggested increasing the length of time between the start of the intervention in adjacent sequences. For example, instead of having the intervention rolled out every time interval, this can be increased to three or more intervals, which can further reduce the impact of coincidental events, and ensure the observed effects is attributed to the interventions (Slocum et al., 2022).

### Statistical model

In this section, we introduce statistical models that can analyze data from an MBFD.

### MBFD without interaction effect (Model 1)

In a standard MBFD, each participant is followed over time and their outcomes are measured repeatedly. Let us assume that we have $N$ participants, and each one contributes $J$ observations. Let ${Y}_{ij}$ denote the continuous outcome of $i-th$ individual at time $j$. Under the assumption of conditional independence (given random effects), the data can be modeled as, ${Y}_{ij}$ can be modeled as

1 $${Y}_{ij}=u+ {\beta }_{T}*j+{\beta }_{A}{X}_{Aij}+{\beta }_{B}{X}_{Bij}+{\alpha }_{i}+{\varepsilon }_{ij}$$

Here, the overall baseline mean is denoted as $u$. ${\beta }_{T}$, a continuous time interval effect, is used to control for the underlying time trend. ${X}_{Aij}$ and ${X}_{Bij}$ are binary indicators for whether individual $i$ received intervention A and/or B at time $j$, taking the value of one when the individual receives the corresponding intervention; otherwise, it is zero. The random intercept term, which links observations for each individual, is expressed by ${\alpha }_{i}$, following a normal distribution $N(0, {\tau }^{2})$. Finally, the residual error term is expressed by ${\varepsilon }_{ij} \sim N(0, {\sigma }^{2})$.

Model 1 assumes no A and B interaction and no history/order (sequence) dependence (i.e., the effect of A does not depend on prior or concurrent exposure to B, and vice-versa). In this longitudinal MBFD, ${\beta }_{A}$ and ${\beta }_{B}$ are parameters for the averaged treatment effects of interventions A and B over the exposed intervals. MBFD staggers the timing of intervention implementation across different sequences, so the baseline and intervention phases are not contemporaneous across each of the sequences (e.g., the baseline and intervention data are not collected simultaneously). This requires us to control for the potential underlying time trends.

During the combined (sequential) phase, ${X}_{Aij}=1$ and ${X}_{Bij}=1$; under the additivity and no sequence and carryover effect assumption, the expected effect of receiving both interventions equals ${\beta }_{A}$ + ${\beta }_{B}$. This assumption may be violated in practice, especially under sequential delivery, so it should be evaluated contextually. If interaction or historical effects are present, estimates of ${\beta }_{A}$, ${\beta }_{B}$, and the implied AB effect will be biased under Model 1.

### MBFD with interaction effect (Model 2)

To relax the assumption of additive effects, we introduce an interaction term between the two interventions:

2 $${Y}_{ij}=u+ {\beta }_{T}*J+{\beta }_{A}{X}_{Aij}+{\beta }_{B}{X}_{Bij}+{\beta }_{I}{X}_{Aij}{X}_{Bij}+{\alpha }_{i}+{\varepsilon }_{ij}$$

In this model, we add a single interaction term ${X}_{Aij}{X}_{Bij}$. Because AB is delivered sequentially, ${\beta }_{I}$ quantifies the departure from additivity under sequential delivery (no sequence effects). That is how the effect of the second intervention changes given prior exposure to the first, which should not be interpreted as a concurrent factorial interaction. If ${\beta }_{I}\ne 0$, the combined treatment effect deviates from the additive sum (${\beta }_{A}$ + ${\beta }_{B}$). Thus, this model allows estimation of whether the effect of one intervention is modified by the presence of the other in sequential delivery.

### Alternative multiple-arm model (Model 3)

As an alternative, the combined intervention can be modeled as a distinct treatment arm:

3 $${Y}_{ij}=u+ {\beta }_{T}*J+{\beta }_{A}{Z}_{Aij}+{\beta }_{B}{Z}_{Bij}+{\beta }_{C}{Z}_{Cij}+{\alpha }_{i}+{\varepsilon }_{ij}$$

In this specification, binary indicators ${Z}_{Aij}$, ${Z}_{Bij}$ and ${Z}_{Cij}$ are mutually exclusive binary indicators for A, B, and the combination AB (denoted as C). The coefficient ${\beta }_{C}$​ represents the average effect of sequential exposure to both interventions vs. SoC under the randomization. The incremental effect of adding the second intervention can be obtained by comparing ${\beta }_{C}$​ with the appropriate main effect (${\beta }_{A}$​ or ${\beta }_{B}$​), averaged across randomization order.

### Estimands

Specifying estimands is essential to interpreting model parameters and aligning them with the study’s clinical or scientific objectives (Kahan et al., 2024). In MBFDs, sequential exposure complicates the interpretation of treatment effects. Drawing from the factorial design literature (Kahan et al., 2022), we highlight three relevant estimands:

- E1: Effect of intervention A (or B) in the absence of the other.
- E2: Effect of intervention A (or B) in the presence of the other.
- E3: Combined effect of interventions A and B when delivered sequentially, following prior exposure to A or B.

To aid interpretation, we provide a structured comparison of the three statistical models considered in this manuscript. Table 1 summarizes the key assumptions, parameters of interest, and how the combined effect of interventions A and B is represented under each approach. In Model 1, the combined effect is assumed to be additive (​${\beta }_{A}$ + ${\beta }_{B}$) and unbiased estimation relies on the absence of interaction or sequence effects. Model 2 relaxes this assumption by including an interaction term, allowing the combined effect to depart from additivity (${\beta }_{A}$ + ${\beta }_{B}$ + ${\beta }_{I}$). Finally, Model 3 treats the combined intervention as a distinct treatment condition, estimating its effect directly (${\beta }_{C}$) without requiring additivity. This structured comparison highlights how each model targets a slightly different estimand and underscores the importance of selecting a model consistent with the trial’s objectives and underlying scientific assumptions. Table 2 shows how each of the estimand match our hypotheses for example trial.

**Table 1:** Estimands in MBFD and corresponding estimates from all models

Model	Key Assumptions	Parameters of Interest	Representation of Combined Effect
Model 1: Additive (no interaction)	- No A×B interaction - No sequence effects - No carryover effect	*β*_*A*_: Average effect of A vs SoC *β*_*B*_: Average effect of B vs SoC	*β*_*A*_ + *β*_*B*_
Model 2: With interaction	- Interaction allowed - No sequence effects	*β*_*A*_: Effect of A vs SoC (without B) *β*_*B*_: Effect of B vs SoC (without A) *β*_*I*_: Departure from additivity under sequential delivery	*β*_*A*_ + *β*_*B*_ + *β*_*I*_
Model 3: Multi-arm specification	- AB treated as a distinct condition - No additivity assumption required - No sequence effects	*β*_*A*_: Effect of A-only vs SoC *β*_*B*_: Effect of B-only vs SoC *β*_*C*_: Effect of AB vs SoC	*β*_*C*_ (can be interested as the averaged effect across orders if sequence effects exist)

**Table 2:** Estimands in MBFD and corresponding estimates for the hypotheses in example trials

Hypothesis	Corresponding estimand	Model(s) for unbiased estimation	Key assumptions
H1: A behavioral intervention to improve adherence alone decreases PTS symptom severity compared to SoC	Effect of A alone vs. SoC (main effect of A in absence of B)	- Model 1 (Additive): ${\beta }_{A}$ estimates effect of A (valid if no interaction or sequence dependence) - Model 2 (Interaction): ${\beta }_{A}$ gives effect of A when B = 0 (directly unbiased even if interaction exists) - Model 3 (Multi-arm): ${\beta }_{A}$ (from A vs. SoC) directly identifies A’s effect	- No carryover beyond modeled terms - For Model 1: assumes no interaction and no sequence effects - For Model 2: unbiased if order effects do not matter (assume on sequence effect)
H2: Pharmacologic management of PTS decreases symptom severity compared to SoC	Effect of B alone vs. SoC (main effect of B in absence of A)	- Model 1 (Additive): ${\beta }_{B}$ estimates effect of B (valid if no interaction or sequence dependence) - Model 2 (Interaction): ${\beta }_{B}$ gives effect of B when A = 0 (robust to interaction) - Model 3 (Multi-arm): ${\beta }_{B}$ (from B vs. SoC) directly estimates B’s effect	- For Model 1: assumes independence (no A–B interaction) - For Model 2: unbiased if no sequence effects
H3: The sequential combination of A and B, following prior exposure to one of them, decreases PTS symptom severity compared to SoC	Effect of A then B (or B then A) vs. SoC (averaged sequential effects)	- Model 1 (Additive): E3 = ${\beta }_{A}+{\beta }_{B}$ (valid only if no interaction/sequence effects) - Model 2 (Interaction): ${\beta }_{A}+{\beta }_{B}+{\beta }_{I}$ gives combined sequential effect (robust if order doesn’t matter) - Model 3 (Multi-arm): ${\beta }_{C}$ directly estimates the AB sequence effect vs. SoC	- For Model 1: requires strict additivity and no sequence dependence - For Model 2: assumes no sequence effects; unbiased if ${\beta }_{I}$ captures modification correctly

### Estimation methods

To obtain estimates from an MBFD, we need to model the correlations between the observations from the same individual but different intervals. Usually, data of this type are modeled using either linear mixed effect model (LMM) or generalized estimating equations (GEE) (Dahmen & Ziegler, 2004; Molenberghs & Verbeke, 2000). Both models can be easily fitted using standard statistical software, such as R, SAS, and Stata. Previous studies have shown that, for continuous outcomes, LMM and GEE produce very similar results (Ouyang et al., 2024).

Models 1 to 3 are examples of LMM. Compared to LMM, GEE uses robust variance estimators that provide reliable estimates even when the random effects are not correctly specified (Gardiner et al., 2009; Liang & Zeger, 1986). For instance, Models 1 to 3 assume that there is only a random intercept with an exchangeable correlation structure, which implies that any two observations from the same individual have the same correlation regardless of when they were measured. However, in real applications, this correlation structure may be more complex, such as auto-regressive (AR) or even unstructured. The actual random effects may also have more components. For example, instead of having random intercept only, the actual random effects may contain both random intercept and slope. In this case, LMM could lead to invalid statistical inferences (Ouyang et al., 2023a, 2024), but GEE would still provide valid estimates in general (Ford & Westgate, 2020). However, in GEE, the classic robust variance estimator tends to underestimate the standard error when the sample size is small. Therefore, a small sample correction is often required in these cases (Ford & Westgate, 2020; Westgate & Burchett, 2016).

### Simulation study

Estimators for the same quantities from different models may have different statistical properties. Therefore, it is critical to evaluate these properties to make recommendations about the appropriate model to use for MBFD. We conducted a simulation to compare the unbiasedness of estimators, the size, and power of hypothesis tests in the context of the MBFD. Specifically, we compared estimates from the three proposed models (Model 1, 2, and 3) using two different analytical approaches (LMM and GEE).

### Data generation

In our simulation study, we modeled data using the standard MBFD structure illustrated in Fig. 1. Participants were individually randomized to one of six intervention sequences. Each individual was observed over five intervals, beginning with a control condition, followed by either intervention A or B, and finally the combined intervention (A + B). The outcome data were generated from Model 3 by assuming that the baseline means ($u$) was zero, and the linear time effect (${\beta }_{T}$) was one. Without loss of generalizability, we assumed $\sigma$ equals one. To reflect a range of plausible within-individual correlations, we varied the random intercept standard deviation ($\tau$) to achieve intraclass correlation coefficients (ICC) of 0.05, 0.10, and 0.30. These correspond to $\in \{0.23, 0.33, 0.65\}$, respectively. Three treatment effect scenarios were considered: (1) null effects $[{\beta }_{A}$, ${\beta }_{B}$, ${\beta }_{C}]={[0.0, 0.0, 0.0]}^{T}$, (2) additive effects (no interaction): $[{\beta }_{A}$, ${\beta }_{B}$, ${\beta }_{C}]={[0.8, 0.8, 1.6]}^{T}$ or (3) non-additive effects (with interaction): ${[0.8, 0.8, 2.0]}^{T}$. The magnitude of the effects was deliberately set to be large. This ensures high statistical power in some conditions, allowing for comparisons of power between the LMM, GEE, and GEE-MD models. If the effects were too small, power would be universally low, making it impossible to discern differences in the relative efficiency of the methods.

To examine how model performance varies with study size, we considered three sample sizes: $N$ = 30, 60, and 120. These values were selected to reflect standard, large, and extremely large sample sizes in rare disease trials. Specifically, $N$ = 30 represents a sample size for single-site rare disease trials, $N$= 60 reflects a multi-site study for individually randomized MBFDs in rare disease, and $N$ = 120 provides insight into performance as statistical power increases and estimation becomes more stable. By simulating across this range, we aimed to assess how bias, type I error, and power behave under realistic constraints, as well as under more favorable conditions where larger sample sizes are available. This helps clarify the strengths and limitations of MBFDs and the corresponding statistical models across a spectrum of practical applications.

### Estimands

We were primarily interested in the effect of interventions A and B in the absence of the other intervention, which can be directly estimated by the beta coefficients (${\beta }_{A}$ or ${\beta }_{B}$) from three models. We presented results for the other parameters (${\beta }_{I}$ or ${\beta }_{C}$) for completeness.

### Data analysis

We used both LMM and GEE to analyze the simulated trial data. Three LMM, shown as Models 1, 2, and 3, were fitted using “lmerTest” package in R (Kuznetsova et al., 2020). Satterthwaite degree of freedom is used in LMM by default. Additionally, three GEE models were also fitted to each simulated dataset using “geepack” package in R (Højsgaard et al., 2024). Degrees of freedom equal to the number of individuals minus the number of parameters in the model were used. These GEE models have the same marginal component as Models 1, 2, and 3 with an exchangeable correlation structure. Since our sample size is as low as 30, we fitted both standard GEE and GEE with Mancl and DeRouen variance estimator (GEE-MD) to correct the biased estimated standard error in GEE when the sample size is small (Mancl & DeRouen, 2001).

### Performance measures

To evaluate the models, we focus on three performance measures, bias, type I error and power.

To estimate bias, we extract the estimated value for parameters of interest and compare it with the true value. Assuming $\beta$ is the parameter of interest, the bias can be estimated as $\frac{1}{{n}_{sim}}{\sum }_{i=1}^{{n}_{sim}}(\widehat{{\beta }_{i}}-\beta )$ (where ${n}_{sim}$ is the number of simulations) (Morris et al., 2019).

To determine the type I error rates, we used the hypothetical trial data simulated under null ($[{\beta }_{A}$, ${\beta }_{B}$, ${\beta }_{C}]={[0, 0, 0]}^{T}$). This enabled us to test three null hypotheses under three models (Model 1: ${{\boldsymbol{\eta}}}_{1}=[{\beta }_{A}$, ${\beta }_{B}]$=${[0, 0]}^{T}$; Model 2: ${{\boldsymbol{\eta}}}_{2}=[{\beta }_{A}$, ${\beta }_{B}$, ${\beta }_{I}]$=${[0, 0, 0]}^{T}$; Model 3: ${{\boldsymbol{\eta}}}_{3}= [{\beta }_{A}$, ${\beta }_{B}$, ${\beta }_{C}]$=${[0, 0, 0]}^{T}$). Then, we calculated the percentage of simulations that falsely rejected the null hypothesis. We used a type I error rate of 0.05 and Bonferroni corrections were applied (e.g., 0.05/2 for Model 1 and 0.05/3 for Models 2 and 3).

To determine power, we used hypothetical trial data simulated under alternative hypothesis,either $[{\beta }_{A}$, ${\beta }_{B}$, ${\beta }_{C}]={[0.8, 0.8, 1.6]}^{T}$(without interaction effect) or ${[0.8, 0.8, 2.0]}^{T}$(with interaction effect). The same LMM and GEE models were fitted, and the power was calculated as the percentage of simulations in which at least one null hypothesis was correctly rejected at the Bonferroni-corrected type I error level.

### Simulation results

Table 3 presents the empirical type I error rates for LMM, standard GEE, and GEE with the Mancl & DeRouen small-sample correction (GEE-MD). The evaluation was conducted across various sample sizes ($N$), intraclass correlation coefficients (ICC), and the three models we reviewed. The LMM has slightly inflated type I error rates $(\alpha )$ but close to nominal level for most conditions.. For Model 1 ($\alpha$= 0.025), error rates were stable and ranged from 0.023 to 0.031. For the more complex Models 2 and 3 ($\alpha$≈ 0.0167), the rates were appropriately lower, ranging from 0.016 to 0.020. The performance of the LMM was robust to changes in both sample size and ICC. The standard GEE approach showed inflation of the type I error rate, particularly at the smallest sample size ($N$= 30). For example, under Model 1 with $N$ = 30, the GEE error rate was as high as 0.047 (nominal $\alpha$ = 0.025), nearly double the target level. This liberal tendency was mitigated as the sample size increased, demonstrating the asymptotic nature of the method. For $N$ = 120, the error rates, while still slightly elevated (e.g., 0.031), approached the nominal level. The application of the MD correction was highly effective in controlling the type I error of the GEE model. The corrected GEE produced error rates that were very similar to the LMM and were consistently close to the nominal alpha. For the condition where the standard GEE was most liberal (Model 1, $N$ = 30), the MD corrected GEE had a much-improved error rate of 0.028. This demonstrates its utility in small-sample contexts.

**Table 3:** Type I error (Bonferroni adjusted) of all models fitted with both linear mixed effect model (LMM) and generating estimating equations (GEE) under various intra-cluster correlation coefficients

ICC	Details		Linear mixed model (LMM)				Generalized estimating equations (GEE)				GEE (Mancl & DeRouen)			
	model	*N*	${\beta }_{A}$	${\beta }_{B}$	${\beta }_{I}$	${\beta }_{C}$	${\beta }_{A}$	${\beta }_{B}$	${\beta }_{I}$	${\beta }_{C}$	${\beta }_{A}$	${\beta }_{B}$	${\beta }_{I}$	${\beta }_{C}$
ICC = 0.05	1	30	0.031	0.026			0.047	0.042			0.028	0.023		
	1	60	0.028	0.030			0.039	0.039			0.028	0.031		
	1	120	0.029	0.023			0.031	0.028			0.027	0.023		
	2	30	0.020	0.019	0.018		0.035	0.034	0.029		0.017	0.018	0.015	
	2	60	0.017	0.020	0.014		0.025	0.028	0.020		0.017	0.018	0.014	
	2	120	0.016	0.016	0.017		0.021	0.021	0.019		0.017	0.017	0.016	
	3	30	0.020	0.019		0.019	0.035	0.034		0.036	0.017	0.018		0.019
	3	60	0.017	0.020		0.019	0.025	0.028		0.026	0.017	0.018		0.020
	3	120	0.016	0.016		0.020	0.021	0.021		0.023	0.017	0.017		0.019
ICC = 0.10	1	30	0.030	0.027			0.047	0.042			0.028	0.023		
	1	60	0.028	0.030			0.036	0.038			0.026	0.029		
	1	120	0.028	0.025			0.032	0.028			0.027	0.025		
	2	30	0.020	0.019	0.018		0.035	0.034	0.029		0.018	0.018	0.015	
	2	60	0.018	0.019	0.014		0.026	0.025	0.020		0.018	0.019	0.014	
	2	120	0.017	0.017	0.017		0.021	0.020	0.019		0.016	0.017	0.016	
	3	30	0.020	0.019		0.022	0.035	0.034		0.035	0.018	0.018		0.019
	3	60	0.018	0.019		0.019	0.026	0.025		0.026	0.018	0.019		0.020
	3	120	0.017	0.017		0.018	0.021	0.020		0.019	0.016	0.017		0.017
ICC = 0.30	1	30	0.028	0.024			0.043	0.038			0.025	0.021		
	1	60	0.026	0.029			0.033	0.037			0.024	0.029		
	1	120	0.027	0.025			0.030	0.029			0.026	0.026		
	2	30	0.020	0.020	0.018		0.034	0.035	0.029		0.016	0.018	0.015	
	2	60	0.020	0.018	0.014		0.026	0.024	0.020		0.018	0.017	0.014	
	2	120	0.017	0.016	0.017		0.020	0.020	0.019		0.016	0.017	0.016	
	3	30	0.020	0.020		0.021	0.034	0.035		0.032	0.016	0.018		0.017
	3	60	0.020	0.018		0.018	0.026	0.024		0.026	0.018	0.017		0.018
	3	120	0.017	0.016		0.017	0.020	0.020		0.017	0.016	0.017		0.014

*N* total number of participants, *ICC* intra-cluster correlation coefficient

When there is no interaction effect between the interventions, all models lead to unbiased estimates. In Table 4, as expected, across all analytical methods, power increased substantially with larger sample sizes and decreased as the strength of the intraclass correlation increased. A crucial trade-off between type I error control and statistical power was observed when comparing the three methods. The standard GEE model consistently produced the highest numerical power, especially at $N$ = 30. For instance, in Model 2 with ICC = 0.30 and $N$= 30, the GEE had a power of 0.511, whereas the LMM and MD-corrected GEE had powers of 0.454 and 0.393, respectively. However, this is a direct consequence of the inflated type I error rate. The LMM and GEE with MD correction, which both demonstrated control of type I error, yielded more conservative (and more accurate) power estimates. The GEE with the MD correction often had the lowest power. As another example, with $N$=30 and ICC = 0.05 (Model 1), the power was 0.740, compared to the LMM’s 0.792. This reflects the statistical “cost” of bias-corrected robust variance estimation in small-sample settings (Table 4).

**Table 4:** Simulated power of detecting the treatment effects among all three models when there is no interaction effect

ICC	Details		Linear mixed model (LMM)				Generalized estimating equations (GEE)				GEE (Mancl & DeRouen)			
	model	*N*	${\beta }_{A}$	${\beta }_{B}$	${\beta }_{I}$	${\beta }_{C}$	${\beta }_{A}$	${\beta }_{B}$	${\beta }_{I}$	${\beta }_{C}$	${\beta }_{A}$	${\beta }_{B}$	${\beta }_{I}$	${\beta }_{C}$
ICC = 0.05	1	30	0.792	0.790			0.818	0.818			0.740	0.741		
	1	60	0.978	0.981			0.979	0.982			0.973	0.976		
	1	120	1.000	1.000			1.000	1.000			1.000	1.000		
	2	30	0.513	0.508	0.018		0.568	0.549	0.029		0.440	0.432	0.015	
	2	60	0.840	0.844	0.014		0.845	0.851	0.020		0.813	0.813	0.014	
	2	120	0.994	0.993	0.017		0.994	0.993	0.019		0.993	0.991	0.016	
	3	30	0.513	0.508		0.972	0.568	0.549		0.976	0.440	0.432		0.952
	3	60	0.840	0.844		1.000	0.845	0.851		1.000	0.813	0.813		1.000
	3	120	0.994	0.993		1.000	0.994	0.993		1.000	0.993	0.991		1.000
ICC = 0.10	1	30	0.775	0.773			0.795	0.796			0.717	0.719		
	1	60	0.971	0.974			0.974	0.977			0.966	0.970		
	1	120	1.000	1.000			1.000	1.000			1.000	1.000		
	2	30	0.494	0.491	0.018		0.552	0.537	0.029		0.430	0.419	0.015	
	2	60	0.827	0.830	0.014		0.837	0.840	0.020		0.795	0.796	0.014	
	2	120	0.992	0.991	0.017		0.993	0.991	0.019		0.992	0.989	0.016	
	3	30	0.494	0.491		0.962	0.552	0.537		0.965	0.430	0.419		0.937
	3	60	0.827	0.830		1.000	0.837	0.840		1.000	0.795	0.796		1.000
	3	120	0.992	0.991		1.000	0.993	0.991		1.000	0.992	0.989		1.000
ICC = 0.30	1	30	0.713	0.714			0.741	0.737			0.657	0.659		
	1	60	0.952	0.954			0.955	0.957			0.943	0.945		
	1	120	0.999	0.999			0.999	0.999			0.999	0.999		
	2	30	0.454	0.452	0.018		0.511	0.503	0.029		0.393	0.378	0.015	
	2	60	0.784	0.786	0.014		0.798	0.802	0.020		0.754	0.754	0.014	
	2	120	0.986	0.984	0.017		0.987	0.985	0.019		0.984	0.980	0.016	
	3	30	0.454	0.452		0.928	0.511	0.503		0.936	0.393	0.378		0.895
	3	60	0.784	0.786		0.998	0.798	0.802		0.998	0.754	0.754		0.998
	3	120	0.986	0.984		1.000	0.987	0.985		1.000	0.984	0.980		1.000

*N* total number of participants, *ICC* intra-cluster correlation coefficient

Table 5 presents the estimated treatment effects from all models in cases where there is an interaction effect, i.e., ${\beta }_{I}$ = 0.40. In this setting, Model 1 incorrectly omits the interaction term and therefore produces substantially biased estimates for the main effects of treatments A and B. Models 2 and 3, which correctly account for the interaction, yield unbiased estimates for all parameters. These findings were consistent regardless of sample size, estimation methods (GEE vs. LMM) or ICC, underscoring that the primary driver of accurate estimation is the correct specification of the model structure.

**Table 5:** Estimates for parameters of interest when there is interaction between intervention A and B

ICC	Details		Linear mixed model (LMM)				Generalized estimating equations (GEE)			
	model	*N*	${\beta }_{A}$	${\beta }_{B}$	${\beta }_{I}$	${\beta }_{C}$	${\beta }_{A}$	${\beta }_{B}$	${\beta }_{I}$	${\beta }_{C}$
ICC = 0.05	1	30	1.005	1.001			1.005	1.001		
	1	60	1.000	1.000			1.000	1.000		
	1	120	0.997	1.001			0.997	1.001		
	2	30	0.807	0.803	0.395		0.807	0.803	0.395	
	2	60	0.799	0.799	0.402		0.799	0.799	0.402	
	2	120	0.799	0.802	0.396		0.799	0.802	0.396	
	3	30	0.807	0.803		2.005	0.807	0.803		2.005
	3	60	0.799	0.799		2.001	0.799	0.799		2.001
	3	120	0.799	0.802		1.998	0.799	0.802		1.998
ICC = 0.10	1	30	1.005	1.001			1.005	1.001		
	1	60	1.000	1.001			1.000	1.001		
	1	120	0.997	1.001			0.997	1.001		
	2	30	0.808	0.804	0.395		0.808	0.804	0.395	
	2	60	0.799	0.800	0.402		0.799	0.800	0.402	
	2	120	0.799	0.802	0.396		0.799	0.802	0.396	
	3	30	0.808	0.804		2.006	0.808	0.804		2.006
	3	60	0.799	0.800		2.001	0.799	0.800		2.001
	3	120	0.799	0.802		1.998	0.799	0.802		1.998
ICC = 0.30	1	30	1.007	1.002			1.007	1.002		
	1	60	1.001	1.001			1.001	1.001		
	1	120	0.998	1.001			0.998	1.001		
	2	30	0.809	0.805	0.395		0.809	0.805	0.395	
	2	60	0.799	0.800	0.402		0.799	0.800	0.402	
	2	120	0.800	0.803	0.396		0.800	0.803	0.396	
	3	30	0.809	0.805		2.009	0.809	0.805		2.008
	3	60	0.799	0.800		2.002	0.799	0.800		2.002
	3	120	0.800	0.803		1.998	0.800	0.803		1.998

*N* total number of participants, *ICC* intra-cluster correlation coefficient

The statistical power to detect the interaction effect was low across all conditions, especially in small samples (Table 6). With a sample size of $N$= 30, power was below 0.11 for all methods. Power increased with sample size but remained modest at $N$=120, reaching about 0.32 to 0.33 for the LMM and GEE methods. This result highlights a well-known challenge. Studies often lack sufficient power to detect true, complex, interaction effects. A critical finding emerges when comparing the power for main effects between the Model 1 (which omits the interaction term) and the correctly specified Model 2. The Model 1 produced increased power for detecting main effects (e.g., 0.937 vs. 0.513 when $N$= 30). This inflation occurs because when a true interaction is ignored, its variance is improperly absorbed by the main effect terms in the model. This makes the main effects artificially large and more statistically significant, leading to an incorrectly high power and potentially erroneous scientific conclusions. It creates an illusion of a strong, simple main effect when the reality is more nuanced.

**Table 6:** Simulated power of detecting the treatment effects among all three models when there is an interaction effect

ICC	Details		Linear mixed model (LMM)				Generalized estimating equations (GEE)				GEE (Mancl & DeRouen)			
	model	*N*	${\beta }_{A}$	${\beta }_{B}$	${\beta }_{I}$	${\beta }_{C}$	${\beta }_{A}$	${\beta }_{B}$	${\beta }_{I}$	${\beta }_{C}$	${\beta }_{A}$	${\beta }_{B}$	${\beta }_{I}$	${\beta }_{C}$
ICC = 0.05	1	30	0.937	0.941			0.941	0.943			0.908	0.907		
	1	60	0.999	0.999			0.999	1.000			0.999	0.999		
	1	120	1.000	1.000			1.000	1.000			1.000	1.000		
	2	30	0.513	0.508	0.076		0.568	0.549	0.107		0.440	0.432	0.057	
	2	60	0.840	0.844	0.151		0.845	0.851	0.170		0.813	0.813	0.133	
	2	120	0.994	0.993	0.317		0.994	0.993	0.331		0.993	0.991	0.303	
	3	30	0.513	0.508		0.999	0.568	0.549		0.998	0.440	0.432		0.994
	3	60	0.840	0.844		1.000	0.845	0.851		1.000	0.813	0.813		1.000
	3	120	0.994	0.993		1.000	0.994	0.993		1.000	0.993	0.991		1.000
ICC = 0.10	1	30	0.927	0.928			0.931	0.932			0.893	0.892		
	1	60	0.999	0.999			0.999	0.999			0.998	0.998		
	1	120	1.000	1.000			1.000	1.000			1.000	1.000		
	2	30	0.494	0.491	0.075		0.552	0.537	0.107		0.430	0.419	0.057	
	2	60	0.827	0.830	0.151		0.837	0.840	0.169		0.795	0.796	0.133	
	2	120	0.992	0.991	0.317		0.993	0.991	0.332		0.992	0.989	0.303	
	3	30	0.494	0.491		0.998	0.552	0.537		0.996	0.430	0.419		0.993
	3	60	0.827	0.830		1.000	0.837	0.840		1.000	0.795	0.796		1.000
	3	120	0.992	0.991		1.000	0.993	0.991		1.000	0.992	0.989		1.000
ICC = 0.30	1	30	0.890	0.892			0.900	0.900			0.851	0.855		
	1	60	0.995	0.997			0.995	0.996			0.994	0.995		
	1	120	1.000	1.000			1.000	1.000			1.000	1.000		
	2	30	0.454	0.452	0.076		0.511	0.503	0.108		0.393	0.378	0.056	
	2	60	0.784	0.786	0.150		0.798	0.802	0.169		0.754	0.754	0.133	
	2	120	0.986	0.984	0.317		0.987	0.985	0.332		0.984	0.980	0.303	
	3	30	0.454	0.452		0.989	0.511	0.503		0.990	0.393	0.378		0.981
	3	60	0.784	0.786		1.000	0.798	0.802		1.000	0.754	0.754		1.000
	3	120	0.986	0.984		1.000	0.987	0.985		1.000	0.984	0.980		1.000

*N* total number of participants, *ICC* intra-cluster correlation coefficient

## Discussion

This article proposed and evaluated a multiple-baseline factorial design that can allow participants to receive all trial interventions, improving retention and reducing ethical concerns, while minimizing sample size requirements. Through simulations, we compared LMM, GEE, and GEE-MD for analyzing data from this design. We demonstrated that Model 1, which omits the interaction term, provides biased main effect estimates when a true interaction is present. As such, while Model 1 provides efficiency gains, we do not recommend its use due to the high risk of misspecification. Either Model 2 or 3 should be used to avoid biased conclusions, unless interactions are implausible. Critically, when performing sample size calculations, a model that accounts for potential interactions should be used to ensure the study is adequately powered for the more realistic scenario.

The primary limitation of the MBFD (for Model 1 and 2) is the potential for carryover or sequence effects, since participants receive a combined treatment (A and B) only after receiving an individual one (A or B). Model 3 attempts to account for this by explicitly modeling main effects, interactions, and the sequential structure. While this approach reduces bias relative to simpler models, it cannot fully eliminate the confounding introduced by the design (e.g., one can only have the averaged effects of AB and BA if sequence effects exist). Therefore, the parameters need to be interpreted based on the assumptions. A standard parallel-group factorial design would avoid sequencing concerns and provide the cleanest, unbiased estimates of both main and interaction effects. Therefore, the proposed MBFD is a pragmatic alternative in specific contexts, such as when participant recruitment is severely limited or when it is important for all individuals to receive all interventions over the course of the study. Whenever it is possible, we would still recommend a standard factorial design.

Our simulations comparing LMM and GEE approaches align with previous research (Westgate & Burchett, 2016). Standard GEE tend to have inflated type I error rates in small sample sizes. In contrast, GEE with the Mancl and DeRouen small-sample correction successfully maintained the nominal error rate. This highlights the necessity of using a small-sample correction for GEE in MFBD. While LMM provided slightly higher statistical power than the GEE-MD approach, the random effects structure must be correctly specified. Given that GEE is robust to the misspecification of the correlation structures, we recommend using GEE with a proven small-sample correction to ensure valid statistical inferences, especially when the number of individuals is limited. 

This study has several limitations. First, our simulation parameters, including the ICC and the magnitude of treatment effects, were chosen for illustrative purposes and were not anchored to specific empirical data. Future applications of this design should use parameters and effect sizes justified by the literature. Second, our investigation of statistical methods was not exhaustive. We focused on the MD correction for GEE, but other methods like the Kenward-Roger correction for LMM or the Kauermann and Carroll correction for GEE warrant investigation (Kauermann & Carroll, 2001; Kenward & Roger, 1997). More recent research showed that a robust variance estimator can work well under LMM for longitudinal and clustered data (Ouyang et al., 2024). Finally, future work could expand the framework to directly compare the combined intervention to individual interventions (e.g., AB vs. A), which is often a key question of interest in factorial trials.

## Conclusion

In this study, we described a novel individually randomized MBFD to investigate two or more interventions against a control or SoC when the sample size is limited. Given the sequential exposure to treatments and the potential confounding from treatment order and carryover, we carefully considered the causal assumptions required to interpret effects, particularly when estimating combined treatment effects. We recommend using this design when the expected recruitment sample size is small and a conventional factorial design cannot provide sufficient power. The estimand of interest must be clarified, and either LMM or GEE, with a small sample correlation, is the most suitable analysis method.

## Data Availability

No data was generated from this study.

## References

[CR1] Avila, L., Amiri, N., De, R., Vincelli, J., Pullenayegum, E., & Brandão, L. R. (2021). Compression garments for the management of pediatric post-thrombotic syndrome: A prospective longitudinal study. *Journal of Thrombosis and Haemostasis*. 10.1111/jth.15507 34418289 10.1111/jth.15507

[CR2] Avila, L., Betensky, M., Cohen, C., Ahuja, S., Goldenberg, N., & Zia, A. (2024). Clinical care of pediatric patients with or at risk of postthrombotic syndrome: Guidance from the ISTH SSC Subcommittee on pediatric and neonatal thrombosis and hemostasis. *Journal of Thrombosis and Haemostasis,**22*(2), 365–378. 10.1016/j.jtha.2023.10.012 37866514 10.1016/j.jtha.2023.10.012 PMC12795625

[CR3] Avila, M. L., Feldman, B. M., Pullenayegum, E., Lumia, C., Montoya, M. I., Vincelli, J., Williams, S., & Brandão, L. R. (2019). Post-thrombotic syndrome in children: Measurement properties of CAPTSure, a new diagnostic tool. *Research and Practice in Thrombosis and Haemostasis,**3*(4), 652–657. 10.1002/rth2.12251 31624784 10.1002/rth2.12251 PMC6781925

[CR4] Avila, M. L., Pullenayegum, E., Williams, S., Yue, N., Krol, P., & Brandão, L. R. (2016). Postthrombotic syndrome and other outcomes of lower extremity deep vein thrombosis in children. *Blood,**128*(14), 1862–1869. 10.1182/blood-2016-03-704585 27474755 10.1182/blood-2016-03-704585

[CR5] Baer, D. M., Wolf, M. M., & Risley, T. R. (1968). Some current dimensions of applied behavior analysis. *Journal of Applied Behavior Analysis,**1*(1), 91–97. 10.1901/jaba.1968.1-91 16795165 10.1901/jaba.1968.1-91 PMC1310980

[CR6] Binik, A. (2019). Delaying and withholding interventions: Ethics and the stepped wedge trial. *Journal of Medical Ethics,**45*(10), 662–667. 10.1136/medethics-2018-105138 31341013 10.1136/medethics-2018-105138

[CR7] Cipriani, A., & Barbui, C. (2013). What is a factorial trial? *Epidemiology and Psychiatric Sciences,**22*(3), 213–215. 10.1017/S2045796013000231 23676770 10.1017/S2045796013000231 PMC8367336

[CR8] Coon, J. C., & Rapp, J. T. (2018). Application of multiple baseline designs in behavior analytic research: Evidence for the influence of new guidelines. *Behavioral Interventions,**33*(2), 160–172. 10.1002/bin.1510

[CR9] Dahmen, G., & Ziegler, A. (2004). Generalized estimating equations in controlled clinical trials: Hypotheses testing. *Biometrical Journal,**46*(2), 214–232. 10.1002/bimj.200310018

[CR10] Dziak, J. J., Nahum-Shani, I., & Collins, L. M. (2012). Multilevel factorial experiments for developing behavioral interventions: Power, sample size, and resource considerations. *Psychological Methods,**17*(2), 153–175. 10.1037/a0026972 22309956 10.1037/a0026972 PMC3351535

[CR11] Epstein, L. H., & Dallery, J. (2022). The Family of Single-Case Experimental Designs. *Harvard Data Science Review*, *Special Issue 3*. 10.1162/99608f92.ff9300a8 10.1162/99608f92.ff9300a8 PMC10016625 36926648

[CR12] Ford, W. P., & Westgate, P. M. (2020). Maintaining the validity of inference in small-sample stepped wedge cluster randomized trials with binary outcomes when using generalized estimating equations. *Statistics in Medicine,**39*(21), 2779–2792. 10.1002/sim.8575 32578264 10.1002/sim.8575

[CR13] Gardiner, J. C., Luo, Z., & Roman, L. A. (2009). Fixed effects, random effects and GEE: What are the differences? *Statistics in Medicine,**28*(2), 221–239. 10.1002/sim.3478 19012297 10.1002/sim.3478

[CR14] Hawkins, N. G., Sanson-Fisher, R. W., Shakeshaft, A., D’Este, C., & Green, L. W. (2007). The multiple baseline design for evaluating population-based research. *American Journal of Preventive Medicine,**33*(2), 162–168. 10.1016/j.amepre.2007.03.020 17673105 10.1016/j.amepre.2007.03.020

[CR15] Hemming, K., Haines, T. P., Chilton, P. J., Girling, A. J., & Lilford, R. J. (2015). The stepped wedge cluster randomised trial: Rationale, design, analysis, and reporting. *BMJ (Clinical research ed.),**350*, Article h391. 10.1136/bmj.h391 25662947 10.1136/bmj.h391

[CR16] Højsgaard, S., Halekoh, U., Yan, J., & Ekstrøm, C. T. (2024). *geepack: Generalized Estimating Equation Package* (Version 1.3.10) [Computer software]. https://cran.r-project.org/web/packages/geepack/index.html

[CR17] Hussey, M. A., & Hughes, J. P. (2007). Design and analysis of stepped wedge cluster randomized trials. *Contemporary Clinical Trials,**28*(2), 182–191. 10.1016/j.cct.2006.05.007 16829207 10.1016/j.cct.2006.05.007

[CR18] Kahan, B. C., Hindley, J., Edwards, M., Cro, S., & Morris, T. P. (2024). The estimands framework: A primer on the ICH E9(R1) addendum. *BMJ,**384*, e076316. 10.1136/bmj-2023-076316 38262663 10.1136/bmj-2023-076316 PMC10802140

[CR19] Kahan, B. C., Morris, T. P., Goulão, B., & Carpenter, J. (2022). Estimands for factorial trials. *Statistics in Medicine,**41*(22), 4299–4310. 10.1002/sim.9510 35751568 10.1002/sim.9510 PMC9542167

[CR20] Kauermann, G., & Carroll, R. J. (2001). A note on the efficiency of sandwich covariance matrix estimation. *Journal of the American Statistical Association,**96*(456), 1387–1396. 10.1198/016214501753382309

[CR21] Kennedy, C. H. (2022). The nonconcurrent multiple-baseline design: It is what it is and not something else. *Perspectives on Behavior Science,**45*(3), 647–650. 10.1007/s40614-022-00343-0 36249167 10.1007/s40614-022-00343-0 PMC9458783

[CR22] Kenward, M. G., & Roger, J. H. (1997). Small sample inference for fixed effects from restricted maximum likelihood. *Biometrics,**53*(3), 983–997. 10.2307/2533558 9333350

[CR23] Krasny-Pacini, A., & Evans, J. (2018). Single-case experimental designs to assess intervention effectiveness in rehabilitation: A practical guide. *Annals of Physical and Rehabilitation Medicine,**61*(3), 164–179. 10.1016/j.rehab.2017.12.002 29253607 10.1016/j.rehab.2017.12.002

[CR24] Kratochwill, T. R., & Levin, J. R. (2010). Enhancing the scientific credibility of single-case intervention research: Randomization to the rescue. *Psychological Methods,**15*(2), 124–144. 10.1037/a0017736 20515235 10.1037/a0017736

[CR25] Kuznetsova, A., Brockhoff, P. B., Christensen, R. H. B., & Jensen, S. P. (2020). *lmerTest: Tests in Linear Mixed Effects Models* (Version 3.1–3) [Computer software]. https://CRAN.R-project.org/package=lmerTest

[CR26] Lanovaz, M. J., & Turgeon, S. (2020). How many tiers do we need? Type I errors and power in multiple baseline designs. *Perspectives on Behavior Science,**43*(3), 605–616. 10.1007/s40614-020-00263-x 33024931 10.1007/s40614-020-00263-x PMC7490309

[CR27] Levin, J. R., & Ferron, J. M. (2021). Different randomized multiple-baseline models for different situations: A practical guide for single-case intervention researchers. *Journal of School Psychology,**86*, 169–177. 10.1016/j.jsp.2021.03.003 34051912 10.1016/j.jsp.2021.03.003

[CR28] Liang, K.-Y., & Zeger, S. L. (1986). Longitudinal data analysis using generalized linear models. *Biometrika,**73*(1), 13–22. 10.2307/2336267

[CR29] Lyons, V. H., Li, L., Hughes, J. P., & Rowhani-Rahbar, A. (2017). Proposed variations of the stepped-wedge design can be used to accommodate multiple interventions. *Journal of Clinical Epidemiology,**86*, 160–167. 10.1016/j.jclinepi.2017.04.004 28412466 10.1016/j.jclinepi.2017.04.004 PMC5835387

[CR30] Mancl, L. A., & DeRouen, T. A. (2001). A covariance estimator for GEE with improved small-sample properties. *Biometrics,**57*(1), 126–134. 10.1111/j.0006-341X.2001.00126.x 11252587 10.1111/j.0006-341x.2001.00126.x

[CR31] Molenberghs, G., & Verbeke, G. (2000). *Linear Mixed Models for Longitudinal Data*. Springer. 10.1007/978-1-4419-0300-6

[CR32] Montoya, M. I., Avila, M. L., Vincelli, J., Williams, S., & Brandão, L. R. (2016). Understanding the barriers in compliance to elastic compression garments in the treatment of pediatric post-thrombotic syndrome: A qualitative study. *Thrombosis Research,**144*, 113–115. 10.1016/j.thromres.2016.06.011 27318248 10.1016/j.thromres.2016.06.011

[CR33] Morris, T. P., White, I. R., & Crowther, M. J. (2019). Using simulation studies to evaluate statistical methods. *Statistics in Medicine,**38*(11), 2074–2102. 10.1002/sim.8086 30652356 10.1002/sim.8086 PMC6492164

[CR34] Mutlak, O., Aslam, M., & Standfield, N. J. (2019). Chronic venous insufficiency: A new concept to understand pathophysiology at the microvascular level – a pilot study. *Perfusion,**34*(1), 84–89. 10.1177/0267659118791682 30067139 10.1177/0267659118791682

[CR35] O’Brien, S. H., Stanek, J. R., Witmer, C. M., & Raffini, L. (2022). The continued rise of venous thromboembolism across US children’s hospitals. *Pediatrics,**149*(3), Article e2021054649. 10.1542/peds.2021-054649 35156127 10.1542/peds.2021-054649

[CR36] Ouyang, Y., Kulkarni, M. A., Protopopoff, N., Li, F., & Taljaard, M. (2023a). Accounting for complex intracluster correlations in longitudinal cluster randomized trials: A case study in malaria vector control. *BMC Medical Research Methodology,**23*(1), 64. 10.1186/s12874-023-01871-2 36932347 10.1186/s12874-023-01871-2 PMC10021932

[CR37] Ouyang, Y., Li, F., Preisser, J. S., & Taljaard, M. (2022). Sample size calculators for planning stepped-wedge cluster randomized trials: A review and comparison. *International Journal of Epidemiology,**51*(6), 2000–2013. 10.1093/ije/dyac123 35679584 10.1093/ije/dyac123 PMC9749719

[CR39] Ouyang, Y., Taljaard, M., Forbes, A. B., & Li, F. (2024). Maintaining the validity of inference from linear mixed models in stepped-wedge cluster randomized trials under misspecified random-effects structures. *Statistical Methods in Medical Research*(9). 10.1177/09622802241248382 10.1177/09622802241248382 PMC11499024 38807552

[CR40] Partington, G., Cro, S., Mason, A., Phillips, R., & Cornelius, V. (2022). Design and analysis features used in small population and rare disease trials: A targeted review. *Journal of Clinical Epidemiology,**144*, 93–101. 10.1016/j.jclinepi.2021.12.009 34910979 10.1016/j.jclinepi.2021.12.009

[CR41] Raffini, L., Huang, Y.-S., Witmer, C., & Feudtner, C. (2009). Dramatic increase in venous thromboembolism in children’s hospitals in the United States from 2001 to 2007. *Pediatrics,**124*(4), 1001–1008. 10.1542/peds.2009-0768 19736261 10.1542/peds.2009-0768

[CR42] Slocum, T. A., Pinkelman, S. E., Joslyn, P. R., & Nichols, B. (2022). Threats to internal validity in multiple-baseline design variations. *Perspectives on Behavior Science,**45*(3), 619–638. 10.1007/s40614-022-00326-1 36249165 10.1007/s40614-022-00326-1 PMC9458807

[CR43] Smith, J. D. (2012). Single-case experimental designs: A systematic review of published research and current standards. *Psychological Methods*. 10.1037/a0029312 22845874 10.1037/a0029312 PMC3652808

[CR44] Sundin, P., & Crespi, C. M. (2022). Power analysis for stepped wedge trials with multiple interventions. *Statistics in Medicine,**41*(8), 1498–1512. 10.1002/sim.9301 35014710 10.1002/sim.9301

[CR45] Watson, P. J., & Workman, E. A. (1981). The non-concurrent multiple baseline across-individuals design: An extension of the traditional multiple baseline design. *Journal of Behavior Therapy and Experimental Psychiatry,**12*(3), 257–259. 10.1016/0005-7916(81)90055-0 7320215 10.1016/0005-7916(81)90055-0

[CR46] Westgate, P. M., & Burchett, W. W. (2016). Improving power in small-sample longitudinal studies when using generalized estimating equations. *Statistics in Medicine,**35*(21), 3733–3744. 10.1002/sim.6967 27090375 10.1002/sim.6967 PMC4965318

